# Supplemental Text Message Support With the National Diabetes Prevention Program: Pragmatic Comparative Effectiveness Trial

**DOI:** 10.2196/15478

**Published:** 2020-06-18

**Authors:** Natalie D Ritchie, Silvia Gutiérrez-Raghunath, Michael Josh Durfee, Henry Fischer

**Affiliations:** 1 Denver Health Denver, CO United States

**Keywords:** eHealth, prediabetes, texting, weight loss

## Abstract

**Background:**

The evidence-based National Diabetes Prevention Program (NDPP) is now widely disseminated, yet strategies to increase its effectiveness are needed, especially for underserved populations. The yearlong program promotes lifestyle changes for weight loss and can be offered in-person, online, via distance learning, or a combination of modalities. Less is known about which delivery features are optimal and may help address disparities in outcomes for subgroups. We previously demonstrated the efficacy of a stand-alone text messaging intervention based on the NDPP (SMS4PreDM) in a randomized controlled trial in a safety net health care system. Upon broader dissemination, we then showed that SMS4PreDM demonstrated high retention and modest weight loss at a relatively low cost, suggesting the potential to improve in-person NDPP delivery.

**Objective:**

In this study, we aim to compare the effectiveness of in-person NDPP classes with and without supplementary SMS4PreDM on attendance and weight loss outcomes to determine whether text messaging can enhance in-person NDPP delivery for a safety net patient population.

**Methods:**

From 2015 to 2017, patients with diabetes risks were identified primarily from provider referrals and enrolled in NDPP classes, SMS4PreDM, or both per their preference and availability. Participants naturally formed three groups: in-person NDPP with SMS4PreDM (n=236), in-person NDPP alone (n=252), and SMS4PreDM alone (n=285). This analysis compares the first two groups to evaluate whether supplemental text messaging may improve in-person NDPP outcomes. Outcomes for SMS4PreDM-only participants were previously reported. NDPP classes followed standard delivery guidelines, including weekly-to-monthly classes over a year. SMS4PreDM delivery included messages promoting lifestyle change and modest weight loss, sent 6 days per week for 12 months. Differences in characteristics between intervention groups were assessed using chi-square and t tests. Differences in NDPP attendance and weight loss outcomes were analyzed with multivariable linear and logistic regressions.

**Results:**

The mean age was 50.4 years (SD 13.9). Out of a total of 488 participants, 76.2% (n=372) were female and 59.0% (n=288) were Hispanic. An additional 17.2% (n=84) were non-Hispanic white and 12.9% (n=63) were non-Hispanic black. A total of 48.4% (n=236) of participants elected to receive supplemental text message support in addition to NDPP classes. Participants who chose supplemental text message support were on average 5.7 (SD 1.2) years younger (*P*<.001) than the 252 participants who preferred in-person classes alone. Relatively more women and Hispanic individuals enrolled in the NDPP with supplemental text messages than in NDPP classes alone, 83.9% (n=198) vs 69.0% (n=174, *P*<.001) and 68.6% (n=162) vs 50.0% (n=126, *P*=.001), respectively. Attendance and weight loss outcomes were comparable between groups.

**Conclusions:**

Despite its appeal among priority populations, supplemental text messaging did not significantly increase attendance and weight loss for the in-person NDPP. Further research is needed to identify optimal strategies to improve the effectiveness of the NDPP.

## Introduction

The Diabetes Prevention Program was a successful clinical trial to prevent type 2 diabetes, showing intensive lifestyle support reduced incidence by 58% [1], with positive effects lasting long-term [2,3]. The lifestyle intervention was translated into the National Diabetes Prevention Program (NDPP) and has been widely disseminated since 2012 [4]. Per delivery standards established by the Centers for Disease Control and Prevention (CDC), the yearlong program can be offered in-person, online, via distance learning, or a combination of modalities [5]. Based on the original trial, the NDPP promotes ≥5% weight loss through diet and physical activity to reduce diabetes risk [5]. Strategies to optimize the NDPP dissemination are needed to achieve this weight loss goal in real-world practice.

For the in-person NDPP, a national study found a promising 4.2% mean weight loss, but early dropout is problematic and limits weight loss for many participants [4]. Further, there are concerning disparities in outcomes for subgroups including racial or ethnic minorities, low-income non-Hispanic whites, and younger adults, who achieve about half of the weight loss of their respective counterparts [4,6,7]. Disparities in weight loss have largely been attributed to limited attendance [4,6]. Virtually delivered programming may be more convenient than in-person classes by removing barriers like lack of transportation and being more attractive to younger individuals, yet shows comparable overall weight loss (mean 4.3%) and is understudied in diverse groups [8]. Less is known about which virtual delivery features may enhance the NDPP outcomes [9], which is important given the performance-based payment models for the program [10,11]. Supplementing virtually delivered interventions with in-person coaching has been shown to support modest improvements in weight loss (mean 4.6%) [8]. Conversely, whether concurrent virtually delivered education and support could improve the effectiveness of the in-person NDPP appears unknown.

We previously demonstrated the efficacy of a stand-alone text messaging intervention based on the NDPP curriculum (SMS4PreDM) in a randomized controlled trial of 163 patients in an urban safety net health care system (Denver Health) [12]. In a pragmatic effectiveness study of wider SMS4PreDM dissemination at Denver Health, we then showed that stand-alone SMS4PreDM was relatively low in cost to deliver (US $100.92 per each of the 285 participants) and demonstrated high retention, albeit with modest weight loss compared to 1233 observation-only controls [13]. Now, to determine whether text messaging can enhance in-person NDPP delivery, we compare effectiveness of the in-person NDPP with and without supplementary SMS4PreDM on attendance and weight loss outcomes.

## Methods

Denver Health serves one-third of Denver, Colorado residents across its network of community- and school-based clinics, specialty care centers, and a Level 1 hospital. The majority of patients are low-income and of minority racial or ethnic backgrounds [14]. From October 2015 to October 2017, Denver Health offered the in-person NDPP, SMS4PreDM, or both to patients at risk for diabetes. New NDPP classes and concurrent SMS4PreDM programming began approximately each quarter, with advance announcements to Denver Health providers about opportunities to refer eligible patients. Per CDC criteria, eligible participants included adults with a BMI≥24 (≥22 if Asian) and with prediabetes (ie, hemoglobin A1c 5.7-6.4), a former diagnosis of gestational diabetes, or a positive score on a diabetes risk questionnaire [15]. Lifestyle coaches then conducted outreach calls to verify eligibility, preference, and availability to enroll in upcoming NDPP classes, SMS4PreDM, or both. As of September 2016, patients who initially selected in-person NDPP classes (with or without SMS4PreDM) were encouraged to first attend an in-person orientation session designed to increase engagement in the NDPP [16]. At these orientation sessions, patients who had not initially enrolled in SMS4PreDM could also opt in at this time. There were no fees or financial incentives to participate in these risk reduction programs.

Participants naturally formed three groups: those receiving (1) in-person NDPP classes with SMS4PreDM (n=236), (2) NDPP classes alone (n=252), and (3) SMS4PreDM alone (n=285). As already noted, outcomes for the SMS4PreDM-only group were previously reported [13]. This study focuses on determining whether supplemental text message support may improve delivery of in-person NDPP classes, thus comparing the first two groups of participants receiving NDPP classes plus SMS4PreDM vs NDPP classes alone (N=488).

The in-person NDPP was conducted following guidelines established by the CDC [15]. Participants were offered a total of 22-25 sessions over 1 year (depending on scheduling). The classes complied with standards to provide at least 16 hour-long group sessions in months 1-6 and a minimum of 6 sessions in months 7-12 [15]. Trained, bilingual lay health educators served as lifestyle coaches and led sessions in English or Spanish. Classes were held at Denver Health’s community clinics and main campus locations. SMS4PreDM participants received messages promoting lifestyle change and modest weight loss, delivered 6 days per week for 12 months, following our published SMS4PreDM methodology [12]. Content followed the NDPP session schedule for concordance with the topics of in-person classes. SMS4PreDM was also available in English and Spanish.

Demographic characteristics and data on diabetes risks were collected from electronic health records and self-report as needed. Attendance outcomes were the percentage of NDPP sessions attended and days of NDPP attendance (1-365), per previous findings that intensity and duration of participation are key drivers of weight loss in the NDPP [4]. Weight loss outcomes were total percent of body weight lost and achieving ≥5% weight loss in the NDPP, based on weights recorded at the first and last sessions attended. Body weight was measured at each NDPP session on a high-capacity, medical-quality scale. Participants were encouraged to wear consistent attire for weight measurements (eg, removing outerwear). Data collection was completed in September 2018.

Differences in characteristics between intervention groups were assessed using chi-square and *t* tests. Differences in NDPP attendance and weight loss outcomes were analyzed with multivariable linear and logistic regression. Covariates included age, gender, and race or ethnicity. We further controlled for effects of attending orientation sessions, as they were previously found to improve the NDPP outcomes [16]. Post hoc analyses explored language as a moderating variable among Hispanic participants, given that Spanish-speaking (vs English-speaking) participants previously had more weight loss in SMS4PreDM alone [12,13] but not in NDPP classes alone [6]. Analyses were completed using SPSS version 22 (IBM Corp). The Colorado Multiple Institutional Review Board approved this program evaluation project, which was not registered as a clinical trial.

## Results

The majority of the 488 participants were female (n=372, 76.2%) and Hispanic (n=288, 59.0%), among whom 67.4% (n=194) preferred Spanish. An additional 17.2% (n=84) were non-Hispanic white and 12.9% (n=63) were non-Hispanic black. Mean age was 50.4 years (SD 13.9). Average BMI at enrollment was 34.8 (SD 7.7). Almost half (n=236, 48.4%) of participants elected to receive supplemental text message support while attending in-person NDPP classes, and 252 received NDPP class only.

Relatively more women and Hispanic individuals enrolled in the NDPP with supplemental text messages than in NDPP classes alone, 83.9% (n=198) vs 69.0% (n=174; *P*<.001) and 68.6% (n=162) vs 50.0% (n=126; *P*=.001), respectively. Participants who chose supplemental text message support were on average 5.7 (SD 1.2) years younger (*P*<.001) than those preferring in-person classes alone. There were no other significant differences in demographic characteristics or starting BMI between groups. Over one-third (n=193, 39.5%) of participants in this analysis first attended an orientation session. Those who joined NDPP classes after recruitment from an orientation session were more likely to choose supplemental text messages than patients enrolled via outreach calls alone (n=150, 77.7% vs n=86, 29.2%, *P*<.001).

There were no significant differences in attendance and weight loss outcomes between NDPP participants who received supplemental text messages and those who did not (Table 1). Language did not moderate intervention effectiveness among Hispanic participants, whether for attendance or weight loss outcomes. For example, achieving ≥5% weight loss was not significantly modified by language (*P*=.60).

**Table 1:** National Diabetes Prevention Program outcomes with and without supplemental text message support, N=488^a^.

Outcomes	NDPP^b^ with supplemental text message support (n=236), mean (SE)	NDPP without supplemental text message support (n=252), mean (SE)	*P* value
NDPP sessions attended (%)	38.1 (0.0)	36.8 (0.0)	.68
Number of days in NDPP (1-365)	146.0 (9.8)	134.8 (9.9)	.46
Weight loss in NDPP (%)	2.1 (0.3)	1.7 (0.3)	.37
Achieved ≥5% weight loss in NDPP (%)	44 (18.6)	37 (14.2)	.84

^a^Data are presented as adjusted mean and SE of the mean with multivariable linear regression *P* values for continuous variables and unadjusted frequency (%) with multivariable logistical regression *P* values for categorical variables. Adjusted odds ratio for achieving ≥5% weight loss in NDPP with supplemental text message support is 1.065 (95% CI 0.584-1.944, *P*=.84). Covariates include age, gender, race or ethnicity, and completion of orientation session.

^b^NDPP: National Diabetes Prevention Program.

## Discussion

In a safety net health care system, nearly half of patients with diabetes risks who joined in-person NDPP classes elected to also receive supplemental text message support. Supplemental text message support seemed to appeal relatively more to women, Hispanic individuals, and younger participants than other groups. Despite its appeal among priority populations, supplemental text messaging did not significantly increase attendance and weight loss in the NDPP. The extra costs of supplemental text message support may be unwarranted without sufficiently improved risk reduction. Nonetheless, supplemental text message support did not appear detrimental: participants receiving both in-person and virtual content attended classes equally, despite remote access to content.

Overall, weight loss was limited for in-person NDPP participants in this study, both with and without supplemental text message support (2.1% and 1.7% averages, respectively), when contrasted with the national average of 4.2% weight loss [4]. This may reflect overall challenges of serving a safety net patient population and highlights that additional improvements are needed. Further study to increase effectiveness of text messaging may be indicated given that retention is problematic in yearlong in-person classes, contrasted with high retention previously shown for SMS4PreDM participants. Specifically, a national evaluation showed that the majority of participants complete less than half of the in-person NDPP [4], whereas 91% of participants completed the standalone SMS4PreDM intervention [13]. Standalone SMS4PreDM was associated with only minimal weight loss (≤1%) in real-world practice [13], yet even small amounts of weight loss can be clinically meaningful—each kilogram lost is associated with a 16% reduction in diabetes incidence [17]. Future research may be merited to determine whether participants who discontinue in-person NDPP classes may then benefit from acceptable alternatives such as virtually delivered content. At the same time, developing other strategies to improve the NDPP outcomes appears needed, such as motivational enhancements, identifying and removing participation barriers, and addressing social determinants of health.

Limitations include the nonrandomization in this pragmatic study, which likely contributed to demographic differences between comparator groups; although, analyses controlled for these factors. Additional orientation sessions may have better conveyed the opportunity to receive supplemental text message support than outreach calls alone did; although, we also controlled for attendance to these introductory sessions. Participants who discontinued in-person NDPP classes had continued access to SMS4PreDM content, and the extent to which the continued supplemental text message support may have benefited participants is unknown. Further, we were unable to distinguish participants who meaningfully engaged in supplemental text messaging.

In conclusion, findings suggest that, although many patients at high risk for type 2 diabetes select supplemental text message support, this resource does not increase effectiveness of the in-person NDPP. As such, this study may contribute to knowledge about which dissemination features are important or not for delivery of the NDPP.

## Abbreviations

CDC: Centers for Disease Control and Prevention
NDPP: National Diabetes Prevention Program
